# Chemical Eye Injury Caused by Multiple Ant Bites in a Sadhu: A Report of a Rare Case

**DOI:** 10.7759/cureus.83308

**Published:** 2025-05-01

**Authors:** Takshak Shankar, Poonam Arora, Rinku Meena, Devinder Kumar Lalotra, Amrita Paul

**Affiliations:** 1 Emergency Medicine, All India Institute of Medical Sciences, Rishikesh, Rishikesh, IND

**Keywords:** ant bite marks, ant foreign body, chemical eye injuries, safe removal of ant from eye, social emergency medicine

## Abstract

Ant bites are a common occurrence. Although ocular ant bites are uncommon, they pose unique management challenges due to chemical injuries and the adherence of ants to the conjunctiva through their mandibles. Ascetic Hindu monks, also called sadhus in India, live isolated lives, often have poor nutritional status, and may not regularly access medical facilities. As a result, when they present to the emergency department, there are various challenges, such as a lack of medical history and delayed presentation.

An elderly male sadhu in his 80s arrived at the emergency department with an altered mental status of unknown duration. He had multiple ants adhered to his conjunctivae, with a pH of 4 in the left eye and 4.5 in the right eye. He was diagnosed with chemical eye injury (CEI) from multiple ant bites. He was also found to have septic encephalopathy from a urinary tract infection. He was discharged on day 12, conscious, oriented, and with normal visual acuity. CEIs from ant bites require prompt management. Removal of the ants can be challenging, as they grip the conjunctiva firmly with their mandibles. Emergency care for sadhus is further complicated by their isolation, poor nutrition, and unsanitary living conditions.

## Introduction

In Indian society, ascetic Hindu monks, also called sadhus, hold a revered position as a religious group. These individuals renounce worldly life, often taking vows of poverty, and live a life of isolation [1,2]. The management of these patients poses a significant challenge to the emergency physician. Since most of them don’t access medical facilities regularly, there is no information on their baseline health status or any chronic illnesses. Recognition of medical emergencies is also quite delayed in these patients, as they live a life of isolation. Even when they present to the emergency department, there is limited to no history available. Since they are often dependent on alms for food, their nutritional status is also quite poor. Chemical eye injury (CEI) is an ophthalmic emergency with the potential to cause significant visual impairment, with animal-induced ocular injuries constituting an insufficiently studied public health concern [3,4]. Ant bites to the eye are rare, with very few cases reported in the literature [4,5]. Here, we present a case of an elderly sadhu who was residing alone and presented to the emergency department with altered mental status. The patient was ultimately diagnosed with a CEI due to multiple ant bites, alongside septic encephalopathy due to urinary tract infection.

## Case presentation

**Figure 1:**
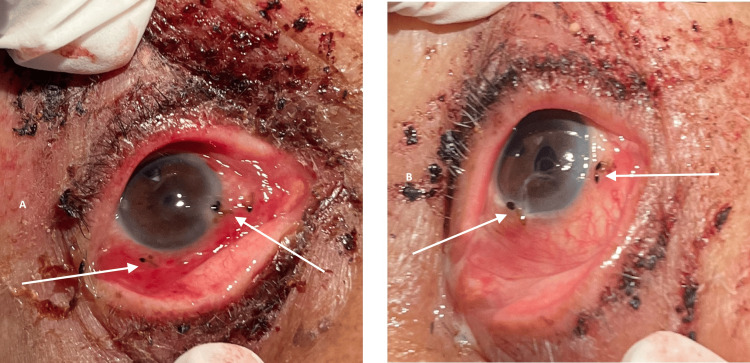
(A, B) Multiple ants adhered to the conjunctiva (indicated by white arrows).

An elderly male sadhu in his early 80s was brought to the emergency department in altered sensorium for an unknown duration. He was found in his cottage lying face down by a group of campers, in a pool of urine and faeces. The campers reported seeing multiple red ants in the cottage. The red ant species could not be clearly identified. On arrival, the patient’s vitals were temperature of 100.6 degrees Fahrenheit, pulse rate of 90 beats/min, blood pressure of 100/60 millimeters of Mercury (mm Hg), peripheral saturation of oxygen (SpO_2_) of 100% on room air, a Glasgow Coma Scale of Eyes3 Verbal1 Motor5 (E3V1M5), random blood sugar of 159 milligrams per deciliter (mg/dL) and a body mass index of 17.6 kilogram per square meter (kg/m^2^). He was moving all four limbs against gravity, and his plantar reflexes were flexor bilaterally. His chest was clear on auscultation, and his abdomen was soft and non-tender. There was no visible limb deformity, and an Extended Focused Assessment with Sonography for Trauma (eFAST) scan was negative. Ocular examination was remarkable for bilateral conjunctival congestion, with 30-50% conjunctival involvement. Both pupils were normal in size and normally reacting to light. Excoriations were noted around the eyelids of bilateral eyes. Multiple ants were found adhered to the conjunctiva through their mandibles (Figure 1).

Visual acuity and visual field examination could not be done owing to the patient’s altered mental status. Anterior segment examination was unremarkable, and the fundus was tessellated. Intraocular pressure was within normal limits. The pH of the left eye was 4, and the right eye was 4.5.

Immediately, the patient’s eyes were irrigated with over three litres of Ringer’s lactate solution till normalization of pH, following instillation of topical anesthesia. While most of the ants were removed through irrigation, some of them remained adhered to the conjunctiva, which were then removed atraumatically through a non-toothed forceps. Eyelids were everted, and conjunctival fornices were examined for any retained foreign bodies. Staining of the anterior segment with fluorescein did not reveal any uptake. He was started on topical antibiotics (ciprofloxacin two drops in both eyes four times a day for five days), along with topical prednisolone (two drops in both eyes three times a day) and artificial tears (carboxymethylcellulose two drops in both eyes four times a day), along with oral vitamin C (500 mg daily). On further evaluation, his altered mental status was attributed to septic encephalopathy secondary to sepsis from an *Escherichia coli* infection of his urinary tract. He was managed with culture-specific antibiotics for the same. The patient responded well to treatment and was discharged on day 12, conscious, oriented, with stable hemodynamics and normal visual acuity using the Snellen chart in both eyes. A written, informed consent to participate was obtained from the patient.

## Discussion

Ants are ubiquitous, making ant bites a very common occurrence. Most of these cases are innocuous, resolving spontaneously. However, severe allergic reactions to ant bites are been increasingly reported [6]. Ant venoms are complex and heterogeneous, with some genera producing non-proteinaceous venoms, comprising formic acid and alkaloids, while other genera produce peptide and protein-rich venoms. These ant venoms possess a myriad of properties, including cytolytic, haemolytic, pro-inflammatory, and allergenic properties. Ants can be both stinging and stingless types. The stinging species use a stinger to inject their secretions, whereas the stingless ones spray their venom. More than half of the venom proteins are allergenic, leading to anaphylactic shock in severe cases [7-9].

While anaphylactic reactions are the reason most patients seek emergency care, ant venom can also lead to CEIs [5]. If a CEI is suspected, the open eyes should be copiously irrigated with a sterile neutral solution. Copious irrigation with water for at least 30 minutes is recommended, although physiologically equivalent irrigating solutions, such as Ringer’s lactate solution, are preferred, as they cause less corneal edema. Topical anesthetic application helps to relieve pain and blepharospasm, and thus helps the patient to keep his eyes open. pH of both eyes should be assessed, both before irrigation as well as after irrigation, to assess adequacy of irrigation [3,10].

Potent topical corticosteroids, such as dexamethasone 0.1%, are recommended to control the acute inflammatory reaction that results from CEI. Topical antibiotics are recommended for the prophylaxis and treatment of infections. Treatment with anti-proteases such as tetracycline, acetylcysteine, and sodium citrate may help in preventing or decreasing the corneal ulceration following CEI. Healing of the damaged ocular surface after CEI is facilitated through various measures such as preservative-free artificial tears and ascorbic acid. Mydriatic and cycloplegic agents are used for the treatment of iridocyclitis. They may also help to prevent or break synechiae [3,11,12]. Ascorbic acid has multiple beneficial effects in patients with CEI. It not only helps to restore the levels of ascorbic acid in the aqueous humour but also reduces degradation, ulceration, and perforation of the cornea [13].

Ants are often attached to the conjunctiva through their mandibles, making their removal difficult. Although there are no defined guidelines for the removal of ants adherent to the conjunctiva, previously published literature describes the usage of a cotton-tipped applicator or a non-toothed forceps to remove the ant, or the usage of a bent 30-gauge needle to gently manipulate the mobile conjunctiva underlying the mandibles of the ants, facilitating their removal [5].

Our patient was an elderly sadhu, residing alone, who presented to the emergency department in altered mental status. There was no history available. Since the patient was living alone, in unclean conditions, he was likely attacked by ants around the eyes when he became unresponsive, complicating his medical condition.

## Conclusions

CEIs should be suspected in all cases of ant bites and must be managed promptly and appropriately. Furthermore, it is crucial to remain vigilant for ocular involvement in patients presenting with altered sensorium. The removal of ants from the conjunctiva can be particularly challenging due to their firm attachment via mandibles. For sadhus, factors such as a solitary lifestyle, poor nutritional status, and unsanitary living conditions further complicate emergency care. These social and environmental determinants play a significant role in shaping the clinical presentation and management of patients in emergency settings.
